# Chagas disease-induced ventricular tachycardia: A case report

**DOI:** 10.21542/gcsp.2023.3

**Published:** 2023-01-30

**Authors:** Zaineb Alhassani, Hussam Al Hennawi, Prerana Sevella, Carolina Severiche Mena, Kathleen Coppola

**Affiliations:** 1 Department of Internal Medicine, Jefferson Abington Hospital, Abington, PA, USA

## Abstract

Chagas disease is a protozoal infection caused by Trypanosoma cruzi (*T. cruzi*) that can affect many organ systems. Chagas cardiomyopathy tends to affect 30% of infected individuals. Cardiac manifestations include myocardial fibrosis, conduction defects, cardiomyopathy, ventricular tachycardia, and sudden cardiac death. In this report, we discuss a 51-year-old male who presented with recurrent episodes of non-sustained ventricular tachycardia refractory to medical therapy.

## Introduction

Chagas disease is a vector-borne tropical/subtropical parasitic disease endemic to the Continental Western hemisphere^1^. This disease is caused by the protozoan Trypanosoma cruzi and transmitted by infected triatomine bugs, which are historically highly prevalent in rural areas of Latin America^2,3^. In non-endemic areas, vertical transmission from mother to fetus, ingestion of contaminated food or drink, blood transfusion, and organ transplant from an infected donor have also been reported as methods of infection^4,5^. Exceptionally, transmission after a laboratory accident has also been described^6^.

Chagas disease is characterized by an acute phase (8–12 weeks) followed by a chronic phase (indeterminate vs. determinate disease). Most patients remain asymptomatic and unaware of the disease. In the acute phase, some patients may experience mild, nonspecific symptoms, the Romaña sign (eyelid swelling), or chagomas (skin abscess). Approximately 30–40% of patients develop chronic disease characterized by megacolon and megaesophagus, cardiomyopathy, arrhythmias, and more rarely, polyneuropathy and stroke^2,7,8^. The cardiac form is the most frequent manifestation of chronic disease and affects around 20%–30% of affected individuals. Chronic Chagas cardiomyopathy (CCC) ranges from a minimal symptomatic disease to severe illness and premature death, and it is usually biventricular with a predominance of right-sided heart failure (lower extremity edema, hepatomegaly, etc.)^8,9^. Treatment remains marginally effective, and antitrypanosomal therapy is recommended only for patients with AHA/ACC stages A and B heart failure. Based on the multicenter, double-blind BENEFIT trial results, antitrypanosomal therapy is not recommended for patients with AHA/ACC stage C or D heart failure^10^.

### Case report

A 51-year-old Brazilian male patient with a history of hypertension, hyperlipidemia, Chagas disease, and chronic pericardial effusion presented to the emergency room complaining of chest pain for three days associated with lightheadedness, shortness of breath, and fatigue. The patient had similar symptoms 2 months prior to presentation, with no evidence of ischemia on a cardiac catheter. On admission, the patient was alert and oriented with a blood pressure of 134/93 mmHg, pulse of 96 beats per minute, temperature of 37.3 °C, respiratory rate of 20 breaths per minute, and oxygen saturation of 97% on room air.

**Figure 1.**
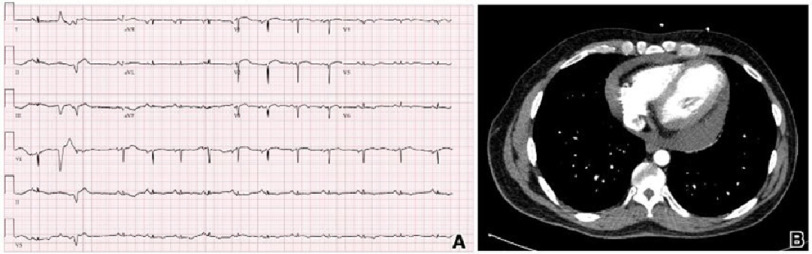
Initial electrocardiogram demonstrated normal sinus rhythm with occasional premature ventricular contractures and non-specific ST/T wave changes. Chest computed tomography with contrast revealed moderate pericardial effusion.

**Figure 2.**
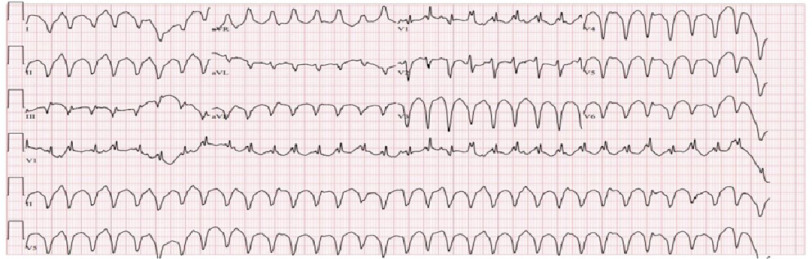
A subsequent electrocardiogram demonstrated wide QRS complex tachycardia consistent with sustained ventricular tachycardia.

Electrocardiography revealed normal sinus rhythm with premature ventricular contractions and non-specific ST/T wave changes (Figure 1A). Lab results were normal except for troponin 20 ng/mL, NT-Pro BNP 268 pg/mL. Chest radiography revealed no abnormal findings. Computed tomography (CT) with contrast of the chest revealed moderate to large pericardial effusion (Figure 1B). The patient developed ventricular tachycardia in the ER (Figure 2).

The patient was instructed to perform the Valsalva maneuver; the rhythm broke and recurred. A repeat electrocardiogram (EKG) was concerning for supraventricular tachycardia (SVT). Adenosine (6 mg) was administered. The episodes of ventricular tachycardia recurred. Thereafter, the patient was admitted to the cardiology care unit with a concern for ventricular tachycardia (VT) storm, defined as persistent or recurrent VT within a 24-hour period.

Antiarrhythmic therapy with intravenous (IV) amiodarone was initiated along with metoprolol. Transthoracic echocardiography revealed an ejection fraction (EF) of 35–40%, along with apical akinesis, moderate inferior hypokinesis, possible aneurysmal apex, and moderate-to-large pericardial effusion with no tamponade physiology. No left ventricular (LV) thrombus was noted, and anticoagulation therapy was discontinued. Given the unremarkable atherosclerotic burden from recent cardiac catheterization, the findings were attributed to Chagas cardiomyopathy. The patient was positive for Trypanosoma cruzi antibody.

Amiodarone was discontinued in light of sustained normal sinus rhythm with occasional premature ventricular contractions (PVCs). An implantable cardioverter-defibrillator (ICD) was later implanted, and the patient was deemed stable for discharge. Of note, cardiac magnetic resonant imaging (MRI) performed during admission was pending at the time of discharge.

**Figure 3.**
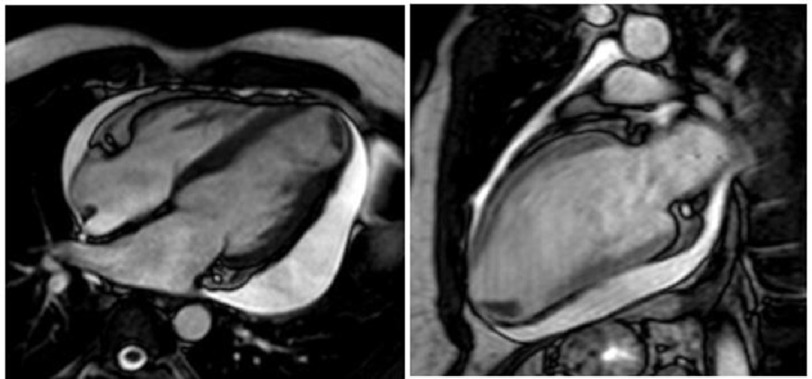
Cardiac MRI revealed a left apical aneurysm with transmural infarction and a left apical thrombus. Epicardial delayed myocardial enhancement in several regions was evident.

In a follow-up appointment, the patient reported feeling better overall, but with continued symptoms of shortness of breath, lightheadedness, and left upper extremity paresthesia. A review of his recent cardiac MRI revealed a left apical thrombus and aneurysm with transmural infarction, consistent with myocardial fibrosis associated with Chagas disease (Figure 3). The patient was ultimately readmitted to the hospital and started on a heparin drip with a bridge to warfarin until a therapeutic INR was achieved. Repeat echocardiography revealed continued pericardial effusion with early signs of tamponade, although this was stable. Pericardiocentesis was deferred. During his stay, the patient experienced multiple runs of ventricular tachycardia. He was started on amiodarone tablets with resolution of arrhythmia and was discharged with close outpatient follow-up.

## Discussion

Chagas cardiomyopathy is a well-known chronic manifestation of Chagas disease. It can be prevented by treating the acute disease with antitrypanosomal treatment or by screening and treating individuals during the asymptomatic phase. Screening may be beneficial in high-risk individuals—people with exposure to the triatomine bug, people born in or living for at least 6 months in an endemic area of Latin America, and especially women of reproductive age in the prenatal or antenatal phases—to avoid congenital transmission. Additionally, organ donors, transplant candidates/recipients, or immunocompromised individuals, such as HIV patients with epidemiological risk factors, should be screened. The IgG serological test is the recommended screening test for individuals 9 months or older. A positive screening test must be followed by a different serological assay for confirmation. Confirmatory diagnosis requires at least two distinct assays based on different antigens^11^. Once confirmed to be in the acute phase of the disease, the patient should be offered antitrypanosomal therapy with a two-month course of benznidazole or a 3–4 month course of nifurtimox. Treatment success can be monitored with PCR or IgG serology.

In untreated individuals, the earliest sign of CCC is conduction disease, including a left anterior fascicular block or right bundle branch block (RBBB). Arrythmias, such as sustained and non-sustained ventricular tachycardia and ventricular fibrillation, are seen due to scar-related re-entry into the cardiac wall. Chronic myocarditis leads to loss of cardiomyocytes and replacement with fibrous tissue, leading to cardiomyopathy, arrhythmias, aneurysm, and increased risk of cardioembolic events^12^. Patients can experience sudden cardiac death due to arrhythmias, particularly sustained VT, which can degenerate into ventricular fibrillation. VT storm, as seen in our patient, is defined as the occurrence of three or more hemodynamically stable ventricular tachyarrhythmias within 24 h. VT storms can be self-terminating but often require antiarrhythmic drugs or device-related therapies. Beta-blockers are also recommended to combat adrenergic surges that frequently occur in ventricular tachyarrhythmias. Catheter ablation can reduce the risk of recurrent VT and is used as adjunctive treatment in patients with structural heart disease with frequent episodes of VT and ICD shocks. Thromboembolic events contribute to stroke and other morbidities in CCC patients. Thromboembolic events were treated according to the guidelines for non-Chagas patients.

Echocardiographic findings can vary from normal wall motion to localized segmental abnormalities, mainly located at the ventricular apex, inferior, and inferolateral walls. As the disease progresses, it may show severely impaired left and right ventricular systolic functions or globally dilated cardiomyopathy. Once cardiomyopathy is diagnosed, the left ventricular diameter and ejection fraction are the strongest predictors of mortality. MRI aids in visualizing cardiac structural changes when echocardiography views are suboptimal. Late gadolinium enhancement can detect changes in the indeterminate stage and is beneficial for clinical disease staging^13,14^. Stress tests and coronary catheterization can be used to distinguish between ischemic cardiomyopathy (ICM) and nonischemic cardiomyopathy (NICM). Endomyocardial biopsy is rarely indicated in patients with suspected CCC since the diagnosis is generally established by other tests.

The mortality of Chagas disease is almost exclusively due to cardiomyopathy; hence, it may be beneficial to stratify the mortality risk in patients with CCC. The major causes of death are sudden cardiac death (55%–65%), progressive HF (25–30%), and stroke (10–15%). Impaired LV function on echocardiography is a common risk factor for death. Others include cardiomegaly on CXR, NSVT on Holter, and NYHA class III/IV.

### What have we learned?

• Chagas disease is caused by infection with protozoan parasite *Trypanosoma cruzi*.
• Disease screening is indicated in patients with a high epidemiologic risk.
• Acute disease may result in mild, nonspecific symptoms, Romaña sign, or chagomas.
• Chronic manifestations include Chagas cardiomyopathy and Chagas gastrointestinal (GI) disease.
• Diagnosis generally involves blood smear or PCR in the acute phase and two serological tests for IgG in the chronic phase.
• The treatment of acute/indeterminate disease involves antitrypanosomal therapy (benznidazole or nifurtimox).
• The treatment of chronic determinate disease depends on specific manifestations.
• Chagas-associated VT can be ablated by medium-energy DC shocks, especially in drug-refractory cases after radiofrequency ablation failure.
• A patient-centered plan and close hospital discharge follow-up in a timely manner are essential for improving patient outcomes.
